# Results of the Quantitative and Qualitative Analysis of Homœpathic Medical Preparations

**Published:** 1857-01

**Authors:** Edward H. Parker

**Affiliations:** New York City


### SELECTIONS.

*Results of the Quantitative and Qualitative Analysis of Homœopathic Medical Preparations.* By EDWARD H. PARKER, M.D. of New York City. Read before the New Hampshire State Medical Society. (From the Transactions.)

During the last two years my attention has been repeatedly called to the drugs used by gentlemen professing to practice medicine "Homœopathically." In consequence of my observations I determined, as opportunity offered, to obtain specimens of the remedies actually used by these practitioners, and sold by various pharmaceutists, and to submit them to an experienced chemist for analysis. This has been done in three instances with the following results:—

The first analysis was made of the contents of two vials, marked respectively *Mercurius Solubilis*, and *Arsenicum*. This is their history:—

A gentleman with whom I had become acquainted in some business connections, often talked to me of his health, and of the treatment to which he was subjected by a prominent Homœopath of this city. Among other powders, he showed me some which he was taking, and which I was sure contained a notable quantity of nitrate of silver. He also praised the treatment to which his child was submitted, when it had a diarrhœa from teething or other cause. A gray powder and a white one were given alternately—and the child liked to take them. His wife kept them constantly by her, and if the child had a discharge from the bowels which she thought was a little too loose, she would give her a few doses of these powders. She thought, however, that the blackish powder (*merc. solub.*) did the most good. My friend constantly urged me to try them; for I think that because I did not rail at Homœopathy, but spoke of its practitioners as I would of other gentlemen, he had some hopes of converting me to his faith, than which I can conceive of no more preposterous supposition. Finally, I requested him to procure for me some of the same powders he was using for his child. This he did, and I placed them in the hands of Dr. Arthur Du Berceau, of this city, who is a skilful analytical chemist. This is his report:—

One hundred parts of white powder, marked *Arsenicum Alb.* contains 1.112 of *Arsenious Acid;* the remainder is *cane* sugar. The second, marked *Solubilis Mercury*, contains in one hundred parts 11.00 of *Metallic Mercury;* the remainder is *cane* sugar. The mercury was in the condition of black oxyd, obtained by the reaction of proto-nitrate of mercury and ammonia.

The mother, when told of the amount of mercury and arsenic which she had been giving to the child, was horrified, and has since used them less indiscriminately.

At my request the same friend purchased for me a case of medicines of a Homœopathic druggist. It is like those which he ordinarily sells for family use. This I also placed in the hands of Dr. Du Berceau, and he obtained the following results:

In the bottle marked Calc. Carb. one hundred parts of powder contain 1.066 Carbonate of Lime.

In the bottle marked Carb. Vegetablis, one hundred parts of powder contain 0.500 fine Charcoal.

In the bottle marked Arsen. Alb. one hundred parts of powder contain 1.120 Arsenious Acid.

In the bottle marked Mercur. Solub. one hundred parts of powder contain 1.350 Metallic Mercury.

In the bottle marked Hepar Sulph. one hundred parts of powder contain 0.900 Sulphur.

In the bottle marked Stibium, one hundred parts of powder contain 0.500 Oxyd of Antimony.

In the bottle marked Sulphur, one hundred parts solution contain 0.100 Sulphur.

In the bottle marked Phosphorus, one hundred parts solution contain 0.430 Phosphorus.

The fluid contents of the vials in the case, with the exception of the last two in the preceding list, were not examined; partly because I wished to preserve them to satisfy the minds of those who might desire to see for themselves; and, partly, because it is so difficult to do anything more than to ascertain the quantity of solid matter which remains after evaporation of the menstruum. The qualitative analysis of organic substances is well known to be one of the most difficult and uncertain of the operations of the chemist. The sugar in these powders was that obtained from milk.

It will be observed, that in this instance the arsenic and soluble mercury are the strongest preparations, though the latter does not compare in its amount of metallic mercury with the proportion found in the first analysis. These two remedies seem to be great favorites with Homœopaths, being frequently prescribed by them. Why this is we now understand.

About the same time, I obtained a set of preparations which had been used by a physician who determined to try his hand at Homœopathy, and took advantage of the position which he occupied in one of the dispensaries of New York to make his experiments. After his resignation, the preparations which he had been using were left in the hands of the apothecary of the institution, and some of them were selected by me for analysis. They were purchased at a different shop from those which were before analyzed, and the direction given was, that, when about two-thirds of the vial in any bottle (they were all solutions) were used, the vial should be filled up with proof-spirits. This will, perhaps, account for some of the variations in the strength of the preparations. It was found that there was, of

Tincture of Silica, in one hundred parts 0.025 of Silica.

Tincture of Hepar Sulph. in one hundred parts 0.050 of Hepar Sulph.

Tincture of Baryta Carbonica, in one hundred parts 1.450 of Carbonate of Baryta.

Tincture of Calc. Carbonica, in one hundred parts 0.500 of Carb. of Lime.

Tincture of Arsenica, in one hundred parts 0.025 of Arsenious Acid.

Tincture of Carb. Vegetablis, in one hundred parts 0.050 of Charcoal.

Tincture of Mercurius Solub. in one hundred parts 0.100 of Solub. Mercury.

Tincture of Lachesis, in one hundred parts 0.025 residue after evaporating the alcohol.

Tincture of Sepia, in one hundred parts 0.025 residue after evaporating the alcohol.

Some of these preparations, as the Baryta Carbonica, contained a thick sediment which carried up the per centage. The other preparations which were left were vegetable, and were therefore excluded from the analysis.

These are all the analyses which I have yet caused to be made, but they are somewhat instructive. The first two preparations were obtained by the direction of a homœopathic practitioner, and one of them, the *merc. sol.* is more than one-tenth pure mercury, the proportion of the oxyd being consequently somewhat greater. The "arsenicum" contains 1.112 parts of arsenious acid, while the usual form in which arsenic is given, viz., Fowler's Solution, contains one-half a grain to each fluid drachm, the dose for an adult being about ten drops.

The second analysis was of drugs sold for "family use," and it is observable that the *arsenicum* is even richer in arsenious acid than the first. The *mercurius* has a much smaller portion of metallic mercury, and yet there is sufficient in it to produce all the effects of this metal when given in small doses. The tinctures accompanying the powders are, so near as I can tell by the ordinary modes of examination, of as great if not greater strength than the corresponding preparations used by physicians. Though contained in small ounce vials their color is marked—the rhus toxicodendron, for instance, being of a deep olive color, as is also the tincture of dulcamara. Ipecacuhana, aconite, arnica, cantharides, all give tinctures of decided color in these small vials. The aconite, indeed, I have used for patients, and find that it produces the same results that ordinarily follow the use of the saturated tincture. Having occasion to use tincture of chamomile, I had some made by a druggist, and filled one of the vials with it. The color of the homœopathic preparation was quite as marked as the other. The tincture of *china*, which being translated means *cinchona*, is a good simple tincture of Peruvian bark.

The third set consists of much weaker preparations, and yet here it is noticeable that, excepting the carbonate of lime and the carbonate of baryta, *merc. solub.* stands highest in its proportion.

If an average is made of the per centages of these three analyses, we shall have this result: for the first 6.056, for the second .745, for the third .250. In contrast with these figures others may be put, showing the per centage of the drug which is left in preparations made according to the directions of Hahnemann for potentizing medicines. The first dilution has in one hundred parts 1 part of the drug. The second dilution has in one hundred parts .01 part of the drug. The third has in every one hundred parts .0001 part of the drug. Beyond this it is not necessary to go; though every one remembers how much stress was and still is laid upon high potentizations, those who use the thirtieth dilution being considered very moderate. The two hundredth is much preferred by some, and yet the weakest preparation of these three classes, obtained from direct sources, is stronger than the second dilution.

It may be asked, how it is that such an abandonment of "potentization" should have occurred among Homœopathists themselves, for these drugs came from their pharmaceutists, from the shops patronized by all the prominent men of that school in this city. The question can be answered only by referring to the positions which they now occupy. If these gentlemen are shown such proofs of the strength of these preparations as these analyses afford, or such as the very appearance of their tinctures gives, they will not for a moment deny that we are correct, or that there is anything in this which is inconsistent with Homœopathy. They will say they are *Homœopathists*, but they are not *Hahnemannists*. O no! not they. How could one be so stupid as to make such a blunder. They believe in the doctrine, *similia similibus curantur*, but they do not find that potentization, as taught by Hahnemann, is borne out by experience. To be sure, this is no more than the whole medical profession has been saying ever since the absurd doctrine was propounded, and it is no more than common sense teaches; but if one suggests this to them, and congratulates them on their returning senses, he gets very little thanks for his trouble. The fact, however, of this entire change of position should be more generally known and appreciated by the profession than it is, so that we may not waste time in assailing a position which has been entirely abandoned. It is safe to attribute any supposed effect of a decillionth of a grain of charcoal to imagination, but it is not quite safe to attribute to the same influence the effects of five drops of saturated tincture of aconite. Under these circumstances it might happen that a Homœopath and a physician would both treat a patient in the same way, their only difference being in their process of reasoning. Both give quinine in intermittent fever; the Homœopath because, as he alleges, it will produce in a healthy person similar symptoms: the physician for the reason that he knows it usually cures the disease; not, as is slanderously reported, because he believes it will produce symptoms unlike intermittent fever. He is no *allo*path. It did in fact happen to a friend of mine to be asked to see a patient who was under the care of a Homœopath, not in consultation with him, but because he was desired to give his opinion whether or not it was safe to trust the patient still longer under the treatment. The disease was typhoid fever, and he found spiritus mindereri and all the usual remedies in ordinary doses, the patient doing very well. He could not but say to the attendant, "If this is Homœopathy, I am a Homœopath." To be sure the physician may write a prescription for *cinchona*, and the Homœopath may write one for *china;* or the one for *hydrargyri oxidi nigri*, and the other for *merc. solub.;* one for *antimony*, and the other for *stibium*, but both mean the same thing, and the patient will receive the same drug.

It is a question of practical interest to the profession to ascertain what there is of good, if any, in Homœopathy. Almost every "new school" enables us to gain some profitable suggestions, which repay the labor of sifting them out of a large mass of chaff. The Hahnemannists have tried experiments in the treatment of diseases with nothing, which we should not have been justified in making, and they have thus taught us something in the natural history of disease. In their progress from infinitesimals to large doses, it has been necessary for them to conceal the change in their medicines, and therefore they have studied the art of giving medicines in the most agreeable, or in the least offensive form, and in this respect we can learn something from Homœopathy. The old school of practitioners, who, when called to a patient's house, seem to make it their first duty to fill it with eight-ounce vials, have not entirely passed away, neither have their abominably-tasting compounds entirely disappeared. Their big bottles, their table-spoonful doses, their nauseous mixtures, have driven and still do drive family after family to Homœopaths, simply because it is not human nature to desire to drink such a mixture as tincture of aloes and assafœtida with castor oil and turpentine in equal parts, a wine-glassful at a time, if almost tasteless water or a sweet powder will accomplish the same good. To doctors, even, when they fall sick, an agreeable draught is preferable to one the very thoughts of which stirs them to their lowest depths.

It is not necessary to point out the mode in which concentrated tinctures can be made to supply the place of less powerful preparations. Neither is it necessary to do more than hint at the frequent desirableness of giving small doses often, rather than a single large draught. A few drops of aconite tincture, in water, is vastly pleasanter than even spiritus mindereri or sweets spirits of nitre. The dose of Norwood's veratrum viride is much pleasanter than infusion or even tincture of digitalis.

But the lesson is more important with reference to powders. For adults, solid substances can usually be given in pill form—but there is no necessity of rolling them in powdered aloes. To this day I cannot rid myself of the remembrance of the disgust with which I used to swallow pills so coated, and with difficulty convince myself that the druggists now use only liquorice or more tasteless powders. Still, for these pills we need not select the most bulky drugs. The active principles of plants, when isolated, aid us in diminishing our pills, and will still more when their powders and properties are fully tested.

Children, however, do not readily swallow pills, and agreeable powders are often a great desideratum while treating them. A child's life may depend on his taking remedies willingly and without compulsion. Thorough trituration of the drug with sugar seems to accomplish this best, especially if (when it is practicable) the doses are divided, but repeated oftener. The Homœopathic Dispensatories direct that powders should be placed upon the tongue and allowed to dissolve, when they are to be washed down with a good draught of water. There is some philosophy in this, for the dissolving sugar first gives the impression to the nerves of taste, and the water washes down the balance almost untasted. In the minds of children, moreover, the first taste seems associated with the fact of taking the powder, while the second and more disagreeable one is not remembered against the dosing. To avail one's self of this fact, it is necessary that the sugar should be reduced to an impalpable powder, otherwise the end is not obtained. If, for instance, ordinary crushed or granulated sugar is used, it will be found that it is not an actual powder, but a mass of more or less complete crystals. On mixing a powder with these, it either falls to the bottom, or, clinging to the crystals, coats them over. In this condition the sugar is less readily dissolved than when in powder, and, in addition, each crystal is covered on its outside with the drug, which is first dissolved and gives its taste to the whole mass. Here, then, is the advantage, and the only one, of the triturations recommended by Hahnemann. —*American Medical Monthly*.

